# Factors Influencing Participation of Adults in Voluntary Medical Male Circumcision in Lindi Region, Tanzania

**DOI:** 10.24248/eahrj.v7i1.715

**Published:** 2023-07-12

**Authors:** Sadock Peter Mathias, Amos Kahwa, Godfather Kimaro, Esther Ngadaya, Hellen Nchagwa, Frank Eric, Sayoki Mfinanga, Thereza Mwombeki, Gibson Kagaruki, Lucy Mwenda, Japhet Smeo, Ntuli Kapologwe, Mary Mathania, Denis Russa

**Affiliations:** a Department of Sociology and Anthropology, University of Dar Es Salaam-Tanzania; b National Institute for Medical Research, Muhimbili Research Centre-Tanzania; c Geita Reginal Referral Hospital - Tanzania; d President's Office, Regional Administration and Local Government Tanzania (PO-RALG)-Tanzania; e St John's University of Tanzania; f Department of Anatomy, Muhimbili University Of Health and Allied Sciences

## Abstract

**Background:**

Voluntary Medical Male Circumcision (VMMC) is a surgical procedure done by a qualified medical personnel using anesthesia. In Tanzania, there is a gap between adult men who are not circumcised and adolescents. This calls for a review of the current situation of VMMC services in the community at large in order to inform policymakers and stakeholders involved in the fight against HIV and other sexually transmitted diseases. The present study explored the factors influencing utilisation of VMMC services among adult males in Lindi Region, Tanzania.

**Materials and methods:**

A cross-sectional study was conducted among adults male (15-49 years). Data were collected by using a structured modified measure evaluation quantitative Version 2 questionnaire using a Tablet/Android device with an Open Data Kit (ODK) application (Google Inc. California, USA).

**Results:**

The socio-demographic set up of the study participants was mainly composed of males less than 30yrs, single, unemployed, with primary education as the highest level of study and of a Muslim faith. Majority of the respondents (92%) recommended circumcision to a male family member who is not yet circumcised. The study showed that improved sexual performance (81%), penile hygiene (97%) and circumcision as a modern civilization (96%) to be the key factors that motivated respondents' utilisation of VMMC services. However, only 20.6% of the respondents could barely say that VMMC is a surgical procedure done by qualified medical personnel under anaesthesia. The major reasons for recommending the utilisation of VMMC services to their family members were the VMMC usefulness in preventing STIs (48.9%), cultural practices and norms (31.5%), improved penile hygiene (17.4%) and religious reasons (2.2%).

**Conclusion:**

VMMC is positively favoured by the local coastal communities of the Lindi region of Tanzania Mainland. Our findings may be inferred to reflect on the other neighbouring regions with similar sociocultural set ups such as Mtwara, Pwani, Rural Dar es Salaam and Tanga and the isles of Unguja and Pemba. Programs addressing VMMC may be well instituted in these local communities with high degree of favourability and success.

## BACKGROUND

Unlike the traditional circumcision practices, Voluntary Medical Male Circumcision (VMMC) is a surgical procedure done by qualified medical personnel under anaesthesia to consenting male adult. It is referred to Medical male circumcision when circumcision is performed for medical indication.^1,2^ Circumcision is one of the oldest surgical procedure performed by humans; it dates back to the prehistoric era and it is still one of the most performed surgical procedure in the world today;^3^ and it has been performed throughout history due to social, cultural, religious and medical reasons. The prevalence of VMMC has been ever changing due to advances in socio-cultural and technological factors. The key motivator for VMMC is prevention against HIV/AIDs.^4^ However, the society may also be motivated by other reasons including prevention against sexually transmitted infections (STIs), and cervical cancer; improved penile hygiene, and social acceptance; peer pressure from friends, family or partners; and perceived improved sexual performance in circumcised men.^5^ Physiologically the skin beneath prepuce (which covers the glans penis in uncircumcised individuals) is so soft that it poses no sufficient barrier to HIV virus. Its removal helps in prevention of female to male vaginal transmission.^6,7^ And the in the same manner it helps in the prevention of other STIs such as Human Papilloma Virus (HPV)- the leading cause of cervical cancer in women in developing countries. The major STIs including Gonorrhoea, Chlamydia and Syphilis, which also increase the HIV infectivity, are substantially reduced by VMMC institution.^8^

It is estimated that worldwide, one in three males is circumcised with almost universal a coverage in some countries.^9^ Voluntary medical male circumcision was launched in 2010 in the fourteen African countries with low circumcision rate and high HIV prevalence which included Ethiopia, Kenya, Uganda, Rwanda, Tanzania, Mozambique, Zambia, Zimbabwe, Malawi, Botswana, Namibia, Lesotho, Swaziland and South Africa.^10,11^ Some of these countries have overall low circumcision rates; for instance, Rwanda has 9%, while other countries have areas with both higher and lower circumcision rates such as Kenya and Tanzania due to socio-cultural, ethnicity and religious differences.^12^WHO recommends medical male circumcision to be integrated in the programs for reducing HIV/AIDS infection rates in high disease burden and low circumcision areas. This is evidenced by clinical trials done in Rakai (Uganda)^13^ Kisumu-Kenya^14^ and Orange Farm (South Africa), which have shown that VMMC reduces the risk of new HIV/AIDS among heterosexual transmission risk by 60%; as well as cervical and penile cancer.^15^ Free VMMC in Tanzania was launched in 2010 in some regions such as Tabora and Njombe; then it was extended to Shinyanga, Simiyu, Mwanza, Mara, Geita, Kagera, Kigoma, Iringa, Mbeya, Ruvuma, Katavi, Singida and Mtwara. Over a decade later, the male circumcision coverage has substantially improved despite some spots where it is in still in low proportions.^16, 17^.

In the years that ensued after the introduction of expanded national VMMC programmes in some regions of mainland Tanzania in 2010, the estimated national prevalence of circumcision among men aged 15-49 has been 67% with Shinyanga region leading at 89% coverage, Njombe (67.3%), Mwanza (66%) and Tabora (62.2%). The regions with lowest circumcision prevalence in Tanzania include Katavi 52.7% Geita(52%), Rukwa (34%) and Simiyu (21%).^18^ The Overall prevalence of HIV in Tanzania is 4.7%, but in some areas with low prevalence of circumcision it is higher than the national average; and male circumcision, particularly VMMC is considered a promising long term approach in the quest to combat the burden of HIV/AIDS.^19^ In some recent campaigns, the Government through President's Office–Ministry of Regional Administration and Local Government (PO-RALG) had aimed to strengthen and scale-up a comprehensive package of quality, safe VMMC services among adolescents and adult men age 10 to 29 years to reach 80% prevalence by 2020 and to integrate Early Infant Male Circumcision (EIMC) services in reproductive and child health (RCH) clinics for sustainability. The present work aimed at exploring factors influencing VMMC utilisation in Lindi region of the coastal regions where traditional and religious circumcisions are practiced alongside limited to no Voluntary Medical Male Circumcision services.^16^Data from this study may help to scale up various VMMC campaigns

## MATERIALS AND METHODS

### Study Design and Setting

A mixed descriptive cross sectional study was conducted with the aim to find out factors influencing utilisation of VMMC services among adult males in Lindi community in Tanzania Mainland.

### Study Population and Sample Size

A quantitative questionnaire was administered to 100 conveniently selected sexually active males in the community due to the limited resources and funding. The participants filled in a written informed consent as per requirement of the study IRB ethical clauses.

### Sampling Technique

Multistage random sampling technique was employed in order to reach geographically dispersed and smaller population and cost and time effectiveness. Simple random sampling was conducted at all levels starting from wards and household level where two individual per household was selected by lottery method. Furthermore, participants were identified at home or at working site.

### Inclusion Criteria

A resident who had stayed in the study area 6 months or more was eligible for the study. Males 15 to 49yrs with full sanity and who consented for the study were enrolled.

### Exclusion Criteria

All those who refused to participate, mentally handicapped individuals and under or over the selected age range were excluded from the study.

### Data Collection Process

Data collection was done electronically using tables/Android devices. Prior to downloading the data from the cloud server, all Android devices were checked to ensure that all completed questionnaires had been uploaded to the server. The consented participants were interviewed using a questionnaire with structured questions based on study objectives. We adopted questionnaire with structured questions to collect information from Men aged 15-49 yrs and above 50yrs. In addition, we used structured questionnaire with open questions to conduct interviews. Each question was addressed accordingly. To eliminate interpretation problems and to maintain precision, the questionnaire was pretested and reviewed accordingly. Interviewers were emphasized on use of simple, clear and understandable Swahili questions and the response was saved in Swahili language. Interviewees were visited in the selected houses/VMMC sites and the process of data collection was done face to face.

### Validity

In order to ensure that data collected for the study were of good quality, the researcher carried out a pretest of the questionnaires and interview guide on a small number of respondents with similar characteristics as that targeted in the study. This helped to identify questions that cannot be answered or those might have ambiguity in the meaning to respondents. Such questions were eliminated or corrected prior to field data collection.

### Reliability

Reliability of instruments was ensured through clear instructions and questions that were asked during the study. The research tools were appraised through triangulation. Different respondents were asked the same question by using different instruments so as to allow the researcher to get information on factors influencing adults' participation in VMMC in Lindi Municipal district, Questionnaires produced the same results on repeated trial, a cut-off point for having knowledge reached by choosing the correct answer and not having knowledge by incorrect response.

### Data Acquisition and Management

Tablets/android devices with an (ODK) Open Data Kit and super recorder application for qualitative data recording, software was installed successfully in the same tablet/android for quantitative data collection, informed consents, question papers, Pen, notebooks, laptops, router/modems for local internet. Quantitative questionnaire was administered to the participants after a written informed consent.

### Data Analysis

Data was entered and analysed by using SPSS software. Data analysed according to each specific objective by computing frequencies, mean, percentages, standard deviation and proportion. Chi-square test was used for categorical data and a *P-value ≤0.01*/taken as statistically significant.

### Ethical Consideration

The ethical clearance was obtained from National Research Committee NIMR/HQ/R.8a/Bollix/3562. A written consent of the participants was sough before enrolment into the research. Furthermore, no invasive procedure or breach of personal privacy was exercised in this questionnaire based research.

## RESULTS

Table 1 shows the socio-demographic characteristics of the 100 study participants aged 16 years and above who participated in the study. Over 50% (n=55) were aged 16 to 29 years, and 7% (n=7) were aged 50 years and above. More than half of the participants 54% (n=54) were single, 38% (n=38) were married. Concerning their highest level of education, 45% (n=45) had attained primary education, 36% (n=36) secondary education and 18% (n=18) beyond secondary education level. The Muslims were 80 % (n=80) and about 71% (n=71) were not employed, while 39% (n=39) were engaged in business and 20% in farming.

**TABLE 1:** Socio-Demographic Characteristics of the Study Participants

	Percent (%)
Age	
16–29 years	55.0
30–39years	30.0
40–49 years	7.0
50 years and above	8.0
Marital status	
Married	38.0
Single	54.0
Separated	2.0
Cohabiting	6.0
What is your religion?	
Roman Catholic	9.0
Lutheran	3.0
Anglican	2.0
Muslim	80.0
Other	6.0
What is your level of education?	
Beyond secondary education	18.0
Secondary education	36.0
Primary education	45.0
No formal education	1.0
What is your occupation? (Main activity)	
Farming	20.0
Business	39.0
Fishing	1.0
Employed	12.0
Other (specify)	28.0
Employment status	
Student	4.0
Employed	25.0
Unemployed	71.0

The socio-demographic distribution show that majority of the participants had the age range 16-29 (55%), were single 54%), of Muslim faith (80%), attained primary education as the highest educational level (45%), were engaged in small business for a living (38%) and not in formal employment (71%). The Pearson Chi square test showed the association between VMMC knowledge and the highest education level for the study participants with primary education at 32.4% (n=11), secondary education at 44.1% (n=15) and above secondary education at 23.5% (n=8). Regarding the basic knowledge of the meaning of VMMC, the proportion who correctly describe it as a surgical procedure done by witch doctor using anaesthesia was 76.5 percent (n=26) and proportion who said it is a surgical procedure done by qualified medical personnel using anaesthesia is 20.6 percent (n=7). The fact whether VMMC reduces the risk of new HIV infection could be stated by 61.8% (n=21) whereas 32.4% (n=11) could not tell.

The results in Table 2 above were obtained by analysing data from a structured a questionnaire administered to the participants during the study. A Pearson Chi-Square test was used to test significance of the proportions of the associations in favour of the most frequent response. These findings indicate that men do not think that VMMC may be useful in the prevention of HIV/AIDS (p=0.914). However, there were many positive findings including VMMC as preventative to other STIs (*p=.41*), improve sexual performance (p=.005), sex enjoyment in men (*p=.35*), improvement of penile hygiene (*p<.001*) and circumcision as an indicator of a civilised person (*p<.001*). There was less knowledge to the participants in respect of the importance of VMMC to the female partner. Majority of men had no idea regarding VMMC a prevention of cervical cancer due to human papilloma virus usually harboured in the male penile prepuce (44%) although not statistically significant; *p=.76*). Further, a substantial number proportion of participants knew nothing about the importance of VMMC in respect of female preference of circumcised men (28%) and female sexual pleasure (25%) although both variables were statistically insignificant at *p=0.62* respectively.

**TABLE 2:** Proportion (%) and Association between Voluntary Medical Male Circumcision with VMMC Perception and Practices

Perception/Practice of VMMC	Yes (%)	No (%)	Don't Know (%)	P-value#
Prevents HIV/AIDS	62.0	36.0	2.0	0.914
Prevention of other STIs	51.0	47.0	2.0	0.410
Prevents cervical cancer to a female partner	28.0	28.0	44.0	0.760
Improved sexual performance	81.0	15.0	4.0	0.005
Circumcised men enjoy sex more	83.0	13.0	4.0	0.350
Circumcision improves penile hygiene	97.0	2.0	1.0	<0.001
Circumcised men are favored by women	70.0	2.0	28.0	0.620
Increase Female sexual pleasure	66.0	1.0	25.0	0.620
Circumcision is an indicator of civilization	96.0	4.0	0.0	<0.001

#In favour of the most frequent response

Table 3 shows the utilisation of VMMC services regarding to age groups. The findings show that, the study participants who recommend circumcision to a male family member who is not yet circumcised under age group 16-49 years is 54.4 percent (n=50), 30-39 years is 29.4 (n=27), 40-49 years is 7.6 percent (n=7), and 50 years and above is 8.7 percent (n=8). Furthermore, with consideration of age groups of study participants, the reasons for recommending circumcision to a male family member who is not yet circumcised, the findings revealed that for prevention of STI's reason 16-29 years is 55.6 percent (n=25), 30-39 years is 33.3 percent (n=15), 40-49 years is 6.7 percent (n=3), and 50 years and above is 4.4 percent (n=2). Community culture and customs reason 16-29 years is 62.1 percent (n=18), 30-39 years is 27.6 percent (n=8), 40-49 years is 6.9 percent (n=2), and 50 years and above is 3.5 percent (n=1). Reason for Improved penile hygiene 16-29 years is 37.5 percent (n=6), 30-39 years is 25.0 percent (n=4), 40-49 years is 6.3 percent (n=1), and 50 years and above is 3.3 percent (n=5). Religious reason 16-29 years is 50 percent (n=1), and 40-49 years is 6.3 percent (n=1).

**TABLE 3:** Utilisation of VMMC Services Among Adult Males (15-49) Years Old

Variable	Age Group in Years				
	16–29 n (%)	30-39 n (%)	40-49 n (%)	≥50 n (%)	Total n (%)
Would you do/or recommend circumcision to a male family member?					
No	5 (62.5)	3 (37.5)	0 (0.0)	0 (0.0)	8 (100.0)
Yes	50 (54.4)	27 (29.4)	7 (7.6)	8 (8.7)	92 (100.0)
If yes, explain the reason					
Prevention of STI's	25 (55.6)	15 (33.3)	3 (6.7)	2 (4.4)	45 (48.9%)
Community culture and customs	18 (62.1)	8 (27.6)	2 (6.9)	1 (3.5)	29 (31.5%)
Improved penile hygiene	6 (37.5)	4 (25.0)	1 (6.3)	5 (3.3)	16 (17.4%)
Religious reason	1 (50.0)	0 (0)	1 (6.3)	0 (0)	2 (2.2%)

## DISCUSSION

In these study males (15-49 years) in Lindi region were assessed in regard to knowledge, perception, practice, utilisation of VMMC services and determination of factors associated with VMMC utilisation among adult males. These data suggested that the factors associated with knowledge in respect with uptake of VMMC included highest education level attained, being able to define VMMC and if VMMC reduce the risk of new HIV infections. These results are consistent with previous findings in which a large number of the respondents underwent circumcision as a motivator for them to prevent HIV, STIs and maintenance of hygiene. Similarly, the same study found that having a higher level of education facilitated the uptake of VMMC entailing the education level was a factor likely to encourage the uptake of VMMC. Being able to define VMMC also was positively associated with uptake of VMMC as evidenced in the same study.^20^ The association between VMMC with perception and practice, the analysis revealed that prevention of STIs, improved sexual performance, circumcised men enjoy sex more than uncircumcised men, circumcision is modern civilization and improved penile hygiene were the factors significantly associated with the uptake of VMMC in the present study. These findings are in agreement with previous studies. In these studies male participants' attitudes were generally positive toward VMMC due to peer pressure, women partners' preferences, STIs prevention and cleanliness as key motivating factors in seeking VMMC.^21, 22^

The majority of the respondents (92%) recommended circumcision to a male family member who is not yet circumcised. The participants expressed the reasons for the recommendation of the utilisation of VMMC services to their family member as due to the prevention of STIs (48.9%), community traditions and customs (31.5%), improved penile hygiene (17.4%) and religious reasons (2.2%). Improved sexual performance, circumcision as an indicator of civilization and penile hygiene were the main factors which determined the utilisation of VMMC services. These finding were consistent with one study based in Malawi in which enhanced sexual pleasure, religious beliefs, proven safety, affordability, confidentiality and being hygienic prompted participants to go for circumcision.^23^

## CONCLUSION

VMMC is positively favoured by the local coastal communities of the Lindi region of Tanzania Mainland. Our findings may be inferred to reflect on the other neighbouring regions with similar sociocultural set ups such as Mtwara, Pwani, Rural Dar es Salaam, Tanga, the isles of Unguja, Pemba and Lakezone regions; Mara, Mwanza, Shinyanga, Simiyu, Geita, Kagera and Kigoma. Programs addressing VMMC may be well instituted in these local communities with high degree of favourability and success.

### Recommendation

Universal VMMC programs need to be initiated in the quest to combat HIV and STIs transmission coupled with VMMC awareness campaigns in some local communities.

### Study Limitations

Funding insufficiency limited our study to one costal region of Lindi, Tanzania. Future studies need to expand to other coastal regions with lower VVMC programs.
